# Room of errors simulation for improving patient safety knowledge, attitudes, and performance confidence among nursing students: a quasi-experimental study

**DOI:** 10.3389/fmed.2026.1931432

**Published:** 2026-08-21

**Authors:** Sunmi Jeon, Minyoung Kim

**Affiliations:** 1 Department of Nursing, Daedong College, Busan, Republic of Korea; 2 Department of Nursing, University of Ulsan, Ulsan, Republic of Korea

**Keywords:** medical errors, nursing students, patient safety education, room of errors simulation, simulation training

## Abstract

**Objectives:**

Patient safety incidents remain an important healthcare concern, and patient safety education is essential for preparing nursing students to recognize and prevent preventable errors in clinical settings. Room of errors simulation is a low-cost and practical educational strategy that can help students identify and respond to patient safety hazards in a realistic environment. This study aimed to develop and implement a room of errors simulation for nursing students preparing for clinical practice and to evaluate its effects on patient safety knowledge, attitudes, and performance confidence.

**Methods:**

This quasi-experimental study used a non-equivalent control group pretest-posttest design. Participants were 66 nursing students from three nursing colleges in South Korea. Patient safety knowledge was measured using Park and Park’s instrument, and patient safety attitudes and performance confidence were measured using an adapted Healthcare Professionals Patient Safety Assessment Curriculum Survey. Data were collected in February and March 2024 and analyzed using SPSS version 24.0.

**Results:**

In the experimental group, scores increased from 9.52 ± 1.88 to 12.52 ± 2.12 for patient safety knowledge, from 4.20 ± 0.30 to 4.67 ± 0.30 for patient safety attitudes, and from 4.18 ± 0.50 to 4.59 ± 0.41 for patient safety performance confidence. In the control group, corresponding scores were 7.93 ± 1.89 to 8.13 ± 1.52, 4.10 ± 0.31 to 3.98 ± 0.35, and 4.00 ± 0.76 to 3.86 ± 0.89, respectively. After adjusting for baseline scores, the experimental group showed significantly higher posttest scores than the control group for patient safety knowledge (*F* = 57.56, *p* < 0.001, partial η^2^ = 0.477), patient safety attitudes (*F* = 77.08, *p* < 0.001, partial η^2^ = 0.550), and patient safety performance confidence (*F* = 21.41, *p* < 0.001, partial η^2^ = 0.254).

**Conclusion:**

Room of errors simulation was associated with significant improvements in patient safety knowledge, attitudes, and performance confidence among nursing students preparing for clinical practice. These findings suggest that room of errors simulation may be a feasible educational strategy for strengthening patient safety education before clinical practice.

## 1 Introduction

Since The Institute of Medicine in the US published a report titled “To Err is Human: Building a Safer Health System,” stating that patient safety accidents (PSAs) cause 98,000 deaths and over 1,000,000 injuries per year, PSAs have become a major topic of interest in healthcare (1, 2). PSAs are currently the third leading cause of death for patients in hospitals in the US (3), and as many as 4 in 100 people die from PSAs in some countries (4).

An analysis of PSAs reported to the Korea Patient Safety Reporting and Learning System between 2016 and 2024 revealed that 41.6% were due to falls while 38.6% were caused by medication errors (5). Thus, PSAs are an especially important problem for nurses, who interact closely with patients (6), particularly given that PSAs frequently occur because of nurses’ mistakes or careless behaviors. Such behaviors and their resulting PSAs can result in diverse patient outcomes, from mild problems to death, as well as cause hospitals and countries to incur economic damages because of high medical costs (7). Therefore, error-free nursing is required based on principles and concepts related to patient safety. Given the importance of PSAs, education on patient safety is essential for nurses (8).

Various educational strategies have been used to strengthen patient safety education among nursing students, including flipped learning, case-based simulation, situational awareness education, and simulation-based learning (8–11). Flipped learning promotes preparatory learning and active classroom engagement, case-based simulation helps students apply patient safety principles to realistic clinical situations, and situational awareness education focuses on recognizing clinical cues and anticipating potential risks (8, 10, 11). Among these approaches, simulation-based learning is particularly relevant to patient safety education because it allows students to apply knowledge, skills, and attitudes in realistic clinical environments (9, 12). Types of simulation include virtual reality, augmented reality, high-fidelity, and low-fidelity (13). Of these, low-fidelity simulation is preferred because it is relatively inexpensive, efficient, and does not require expensive equipment or expert skills. One way to implement low-fidelity simulations is through “rooms of errors” (ROEs), a simulation concept developed at the University of Virginia in which patient safety issues are set up in various hospital environments to be resolved by the participants (14). They are indirect patient safety simulations in which participants enter an error-filled environment and must identify as many issues or hazards as possible. Examples of errors might include an incorrect patient name band, fall-related risk factors, or errors relating to medication (15). ROEs offer valuable hands-on experience in identifying issues and recognizing potential risks to patient safety. As ROEs are based on simple scenarios, and various clinical errors can be set up, they can be implemented easily, even by instructors with minimal experience in developing scenarios and running simulations (16).

ROE scenarios are constructed based on commonly reported PSAs (17). ROE participants could be individuals or teams, but teams are more effective (16, 18, 19). Participants can be diverse, including nursing students and medical students, but also nurses, doctors, and hospital staff (14, 20, 21). In previous studies, participant satisfaction was high, and ROEs were effective at improving situational awareness and patient safety awareness (17). ROEs allow students to integrate theoretical knowledge and prior learning experiences by actively identifying and addressing patient safety errors in realistic clinical scenarios (16). This process may promote experiential learning, clinical reasoning, and reflective thinking during patient safety training. ROEs also stimulate students’ interest, encourage active participation, and may facilitate preparation for safe clinical practice (16). The objectives of patient safety education for nursing students are to acquire patient safety competencies, as well as learn and practice the concept and principles of patient safety (22, 23). The importance of patient safety education is increasingly being recognized by nursing colleges, creating a need for research into effective teaching methods and learning strategies (23). Despite the potential benefits of ROE simulations, evidence remains limited regarding their effects as a supplemental educational strategy for nursing students whose regular curriculum includes basic patient safety content, particularly in relation to patient safety knowledge, attitudes, and performance confidence.

Therefore, this study aimed to develop and implement an ROE simulation as a supplemental patient safety education activity for nursing students from three nursing colleges preparing for clinical practice and to evaluate its effects on patient safety knowledge, attitudes, and performance confidence.

## 2 Materials and methods

### 2.1 Study design

This was a quasi-experimental study with a non-equivalent control group pretest-posttest design.

### 2.2 Study setting and participants

Participants were third-year nursing students preparing for clinical practice. Convenience sampling was used to recruit students from three nursing colleges located in different regions of South Korea with similar curricula. To prevent contamination, participants in the control group were recruited from a nursing college in another city via the department website. The inclusion criteria were as follows: (1) third-year nursing students who had completed theoretical nursing coursework including basic patient safety content and classroom laboratory practical training required before clinical practice; (2) no previous experience with ROE simulation-based education; and (3) no clinical practice experience. Participants who did not complete either the pretest or posttest questionnaire were excluded from the final analysis.

### 2.3 Sample size and participant flow

**Figure 1:**
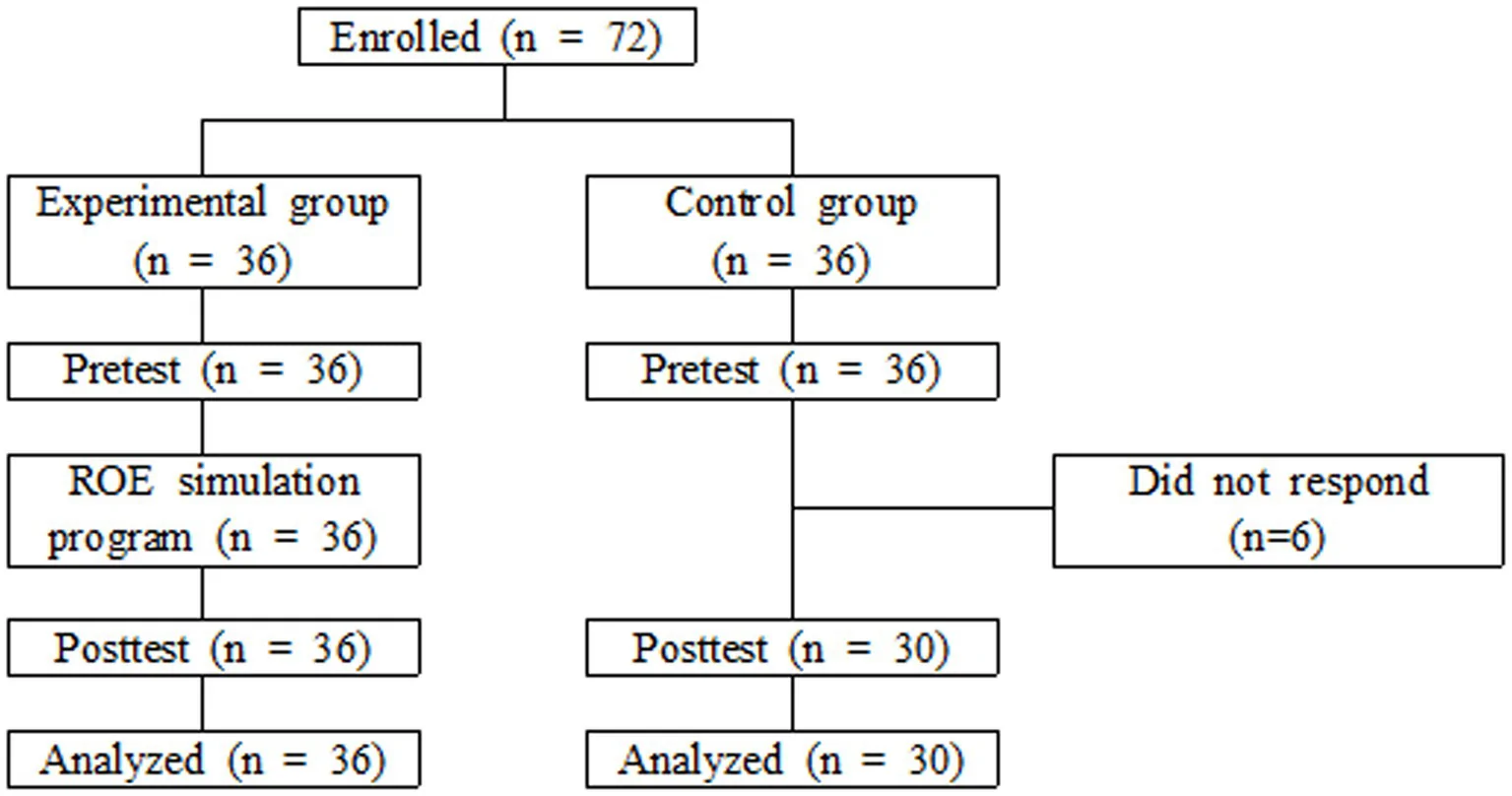
Participant flow diagram.

With reference to previous studies (24), the required sample size was calculated using G*Power version 3.1 (Heinrich Heine University Düsseldorf, Düsseldorf, Germany)^1^ for a two-tailed independent t-test with an effect size of 0.80, a significance level of 0.05, and a power of 0.80. The minimum required sample size was 52. Considering potential attrition, 72 students were recruited, with 36 assigned to each group. Finally, 66 participants were included in the analysis, which exceeded the minimum required sample size. All 36 participants in the experimental group participated in the posttest immediately after the intervention (dropout rate, 0.0%). Of the 36 participants in the control group, 6 did not respond to the invitation to complete the posttest questionnaire despite follow-up contact attempts, resulting in a dropout rate of 16.7%. Thus, data from 66 participants in total (experimental group: 36 participants; control group: 30 participants) were included in the final analysis (Figure 1).

### 2.4 Interventions

#### 2.4.1 Development of the room of errors simulation

For the ROE simulation, we developed a scenario with errors related to patient safety and named it the “Go Out! Room of Errors.” The scenario entailed checking for errors during the hospitalization of a 77-year-old male patient diagnosed with hypertension and diabetes. The medical records provided in advance were progress records, hospitalization records, doctor’s orders, and nursing records.

Based on previous studies (14, 15), patient safety-related errors were divided into 6 categories: physical injury errors, surrounding environment errors, medication errors, patient identification errors, medical waste management errors, and prescription confirmation errors. There were 23 errors in total across these categories (Table 1).

**Table 1:** Staged safety errors in the room of errors simulation.

Item	Category	Staged safety errors
1	Physical injury errors	A needle and alcohol swab in the bed
2		Urine bag being dragged on the floor while the patient has a Foley catheter inserted
3		A Foley catheter wrapped around the bedside rail
4		Bedside rail is not raised
5		Bed wheels are not locked
6		No sign indicating high fall risk for a patient aged 65 years or older
7		Water spilled on the floor around the bed (wet floor for a high-fall-risk patient)
8	Surrounding environment errors	Patient was not wearing their hospital clothes properly (incorrect trouser length)
9		Bedsheets were soiled with patient’s urine
10		Cigarettes and alcohol on the bedside table
11		Use of electronic devices (electric kettle, heated mats, etc.)
12		Hand sanitizer was empty
13	Medication errors	RBC blood pack and fluid bag drips were both attached (duplicate medication)
14		Patient blood type and transfusion blood type did not match
15		Fluids were past their use-by date
16	Patient identification errors	The patient’s name did not match the name on the patient’s chart
17		High fall risk was not indicated on the patient’s ID bracelet
18	Medical waste management errors	A needle was discarded in the general waste box
19		Bloodstained gloves and gauze were discarded in the regular waste basket
20		The ward waste basket was overflowing
21	Prescription confirmation errors	The dose of O2 being provided did not match the prescription
22		The medication being administered did not match the prescription
23		An AST-positive antibiotic (positive drug response test) was prescribed

#### 2.4.2 Intervention

The ROE simulation was run on March 7, 2024, <4 weeks after the pretest. Participants in the experimental group were randomly assigned to eight teams of four to five students each for the intervention, and the intervention took approximately 45 min per team. During a 10-min orientation, the investigators explained the plans for the intervention, precautions during the ROE simulation, and the methods for finding and reporting errors. Thereafter, participants were given 15 min to enter the ROE, find errors related to patient safety, and complete the reporting form. The reporting form included items for identifying and describing patient safety errors observed during the ROE simulation. Team members were allowed to discuss with one another, and one research assistant was situated in the ROE to provide instructions. Finally, there was a 20-min debriefing. During the debriefing session, participants discussed the identified patient safety errors with the investigators, reflected on appropriate patient safety practices, and explored strategies to prevent similar errors in future clinical practice. Participants in the control group did not receive the ROE simulation or any additional research-provided educational intervention during the study period. As with the experimental group, participants in the control group had completed the required theoretical nursing coursework, including basic patient safety content, and classroom laboratory practical training before clinical practice. The control group completed the posttest during the same posttest period as the experimental group. After completion of posttest data collection, the educational content covered in the ROE simulation was shared with participants in the control group, and they were offered an opportunity to participate in the ROE simulation.

### 2.5 Measures

The general characteristics assessed in this study included demographic and academic characteristics: age, sex, academic achievement, satisfaction with the major, previous patient safety education, and experience of patient safety campaigns. Satisfaction with the major was included as an academic characteristic to describe and compare participants’ baseline characteristics between the two groups.

Patient safety knowledge was measured using an instrument developed by Park and Park (25) to examine the extent of patient safety knowledge in nursing students, after receiving permission from the original authors. The instrument was originally developed based on the Korea Institute of Healthcare Accreditation standards and healthcare error-related concepts, thereby reflecting key content areas of patient safety. The instrument comprised 16 questions in total, with 11 questions developed based on the Korea Institute of Healthcare Accreditation, and 5 questions on classification, concepts, and reporting of healthcare errors. The questions were closed, multiple-choice questions, and higher scores indicated greater patient safety knowledge. The Kuder–Richardson Formula 20 (KR-20) coefficient was 0.720 in the original study (25) and 0.634 in this study.

Patient safety attitude and patient safety performance confidence were measured using an adapted version of the Healthcare Professionals Patient Safety Assessment Curriculum Survey (HPPSACS) developed by Chenot and Daniel (26), after obtaining permission from the original authors. The original HPPSACS study included a face validity review and psychometric evaluation, including exploratory factor analysis and internal consistency testing (26). The patient safety attitude instrument consists of 18 questions, scored on a 5-point Likert scale, with higher scores indicating more desirable attitudes toward patient safety. Regarding reliability, the instrument showed Cronbach’s *α* = 0.660 in the study by Chenot and Daniel (26), and Cronbach’s α = 0.852 in our study. The patient safety performance confidence instrument consisted of 5 questions in total, scored on a 5-point Likert scale, with higher scores indicating higher patient safety performance confidence. Regarding reliability, the instrument showed Cronbach’s α = 0.820 in the study by Chenot and Daniel (26) and Cronbach’s α = 0.934 in our study.

### 2.6 Data collection

The pretest was conducted between February 1 and February 11, 2024, using an online questionnaire administered by a research assistant. Participants first completed a consent form and then the pretest questionnaire. Completing the questionnaire took approximately 10 to 15 min.

The posttest was conducted between March 7 and March 9, 2024, using an online questionnaire administered by a research assistant. The experimental group completed the posttest immediately after the ROE simulation intervention to assess the immediate effects of the single-session ROE simulation. The control group, which did not receive the ROE simulation or any additional research-provided educational intervention during the study period, completed the posttest during the same posttest period to maintain comparable timing of outcome assessment and minimize potential contamination between groups. Completing the questionnaire took approximately 10–15 min.

### 2.7 Ethical considerations

This study was approved by the institutional review board at the University of Ulsan (IRB No. 2024R0001-003). The research participation explanation sheet was distributed online to help participants understand the study, and those who voluntarily consented to participate in the study provided written consent. The collected data were stored on a computer that was only accessible to the investigators.

### 2.8 Statistical analysis

The collected data were analyzed using IBM SPSS Statistics version 24.0 (IBM Corp., Armonk, NY, USA). General characteristics were analyzed using frequencies, percentages, means, and standard deviations. Normality of continuous variables was assessed using the Shapiro–Wilk test and visual inspection of Q–Q plots. The independent *t*-test, χ^2^ test, Fisher’s exact test, and Mann–Whitney U test were used to examine homogeneity between the experimental and control groups. Analysis of covariance (ANCOVA) was performed to compare posttest patient safety knowledge, attitude, and performance confidence scores between the groups after adjusting for baseline pretest scores. Before conducting ANCOVA, the assumptions of linearity, homogeneity of variance, homogeneity of regression slopes, and reliability of covariate measurement were examined. Linearity between each baseline pretest score and the corresponding posttest score was assessed using scatterplots. Homogeneity of variance was assessed using Levene’s test. Homogeneity of regression slopes was examined by testing the interaction between group and each baseline pretest score. The reliability of the covariates was assessed using KR-20 for patient safety knowledge and Cronbach’s *α* for patient safety attitudes and performance confidence. Statistical significance was set at *p* < 0.05.

## 3 Results

### 3.1 Participant characteristics

Baseline characteristics and outcome variables were compared between the experimental and control groups. No significant between-group differences were observed in demographic characteristics, patient safety attitude, or patient safety performance confidence. However, baseline patient safety knowledge differed significantly between the groups (Table 2).

**Table 2:** Characteristics of the participants at baseline and comparisons between the groups (*n* = 66).

Variables	Categories	Experimental group (*n* = 36)	Control group (*n* = 30)	*p*
		*n* (%) or Mean ± SD		
Age (years)		21.41 ± 1.59	22.96 ± 5.5	0.145
Gender	Male	3 (8.3)	4 (13.3)	0.693^†^
	Female	33 (91.7)	26 (86.7)	
Academic performance	≥4.0	11 (30.6)	8 (26.4)	0.710^†^
	3.5 ~ 3.9	18 (50.0)	15 (50.0)	
	3.0 ~ 3.4	6 (16.7)	4 (13.3)	
	≤3.0	1 (2.7)	3 (10.0)	
Major satisfaction	High	27 (75.0)	22 (73.3)	1.000^†^
	Moderate	9 (25.0)	8 (26.7)	
	Low	0 (0)	0 (0)	
Patient safety education	Yes	31 (86.1)	27 (90.0)	0.719^†^
	No	5 (13.9)	3 (10.0)	
Patient safety campaign experience	Yes	32 (88.9)	23 (76.7)	0.206^†^
	No	4 (11.1)	7 (23.3)	
Patient safety knowledge		9.52 ± 1.88	7.93 ± 1.89	0.001^‡^
Patient safety attitude		4.20 ± 0.30	4.10 ± 0.31	0.423^‡^
Patient safety performance confidence		4.18 ± 0.50	4.00 ± 0.76	0.254

Values are presented as *n* (%) or mean ± SD. SD, standard deviation. *p*-values were obtained using the independent *t*-test for continuous variables and the *χ*^2^ test for categorical variables unless otherwise indicated. ^†^Fisher’s exact test. ^‡^Mann–Whitney U test.

Table 2 summarizes the characteristics of the participants at baseline and comparisons between the groups (*n* = 66).

### 3.2 Error identification during the room of errors simulation

Table 3 presents the frequency of staged safety errors identified during the room of errors simulation according to the item numbers listed in Table 1. Overall, the identification frequency across all items was 77.2%. At the category level, prescription confirmation errors showed the highest identification frequency (91.7%), followed by physical injury errors (89.3%), medication errors (79.2%), medical waste management errors (79.2%), surrounding environment errors (57.5%), and patient identification errors (56.3%). At the item level, several staged errors were identified by all student teams (100.0%); however, item 8, “patient was not wearing their hospital clothes properly,” and item 9, “bedsheets were soiled with patient’s urine,” had the lowest identification frequency (12.5% each).

**Table 3:** Frequency of staged safety errors identified during the room of errors simulation.

Category	Item number in Table 1	Student teams identifying the error, *n* (%)
Physical injury errors	1	8 (100.0)
	2	8 (100.0)
	3	5 (62.5)
	4	8 (100.0)
	5	8 (100.0)
	6	5 (62.5)
	7	8 (100.0)
Subtotal		**50 (89.3)**
Surrounding environment errors	8	1 (12.5)
	9	1 (12.5)
	10	6 (75.0)
	11	8 (100.0)
	12	7 (87.5)
Subtotal		**23 (57.5)**
Medication errors	13	8 (100.0)
	14	8 (100.0)
	15	3 (37.5)
Subtotal		**19 (79.2)**
Patient identification errors	16	7 (87.5)
	17	2 (25.0)
Subtotal		**9 (56.3)**
Medical waste management errors	18	7 (87.5)
	19	8 (100.0)
	20	4 (50.0)
Subtotal		**19 (79.2)**
Prescription confirmation errors	21	8 (100.0)
	22	8 (100.0)
	23	6 (75.0)
Subtotal		**22 (91.7)**
Total		142 (77.2)

Item numbers correspond to the staged safety errors listed in Table 1. For item-level rows, *n* = 8 student teams in the experimental group. For subtotal and total rows, *n* represents the total possible team-item identifications. Bold values indicate the subtotal for each error category and the overall total.

### 3.3 Efficacy outcomes

Table 4 presents the effects of the ROE simulation on patient safety outcomes among nursing students. Patient safety knowledge scores changed from 9.52 ± 1.88 at pretest to 12.52 ± 2.12 at posttest in the experimental group and from 7.93 ± 1.89 to 8.13 ± 1.52 in the control group. Patient safety attitude scores changed from 4.20 ± 0.30 to 4.67 ± 0.30 in the experimental group and from 4.10 ± 0.31 to 3.98 ± 0.35 in the control group. Patient safety performance confidence scores changed from 4.18 ± 0.50 to 4.59 ± 0.41 in the experimental group and from 4.00 ± 0.76 to 3.86 ± 0.89 in the control group. After adjusting for baseline scores, the experimental group showed significantly higher posttest scores than the control group for patient safety knowledge (*F* = 57.56, *p* < 0.001, partial η^2^ = 0.477), patient safety attitudes (*F* = 77.08, *p* < 0.001, partial η^2^ = 0.550), and patient safety performance confidence (*F* = 21.41, *p* < 0.001, partial η^2^ = 0.254). The partial η^2^ values indicate the proportion of variance in the posttest outcomes explained by group assignment after adjusting for baseline scores.

**Table 4:** Comparison of patient safety knowledge, attitudes, and performance confidence between the experimental and control groups (*n* = 66).

Variables	Groups	Pretest	Posttest	Adjusted	F (*p*)	Partial η^2^
		Mean ± SD		Mean ± SE		
Patient safety knowledge	Experimental	9.52 ± 1.88	12.52 ± 2.12	11.87 ± 0.28	57.56 (<0.001)	0.477
	Control	7.93 ± 1.89	8.13 ± 1.52	8.59 ± 0.31		
Patient safety attitude	Experimental	4.20 ± 0.30	4.67 ± 0.30	4.65 ± 0.05	77.08 (<0.001)	0.550
	Control	4.10 ± 0.31	3.98 ± 0.35	4.01 ± 0.05		
Patient safety performance confidence	Experimental	4.18 ± 0.50	4.59 ± 0.41	4.53 ± 0.09	21.41 (<0.001)	0.254
	Control	4.00 ± 0.76	3.86 ± 0.89	3.93 ± 0.09		

SD, Standard deviation; SE, Standard error.

## 4 Discussion

Our findings showed that nursing students who participated in the ROE simulation demonstrated significantly greater improvements in patient safety knowledge, attitudes, and performance confidence compared to those who did not participate. These findings suggest that engaging students in active identification of patient safety hazards and structured reflection may support the development of competencies required for safe clinical practice.

During the room of errors simulation, the overall identification frequency across all staged safety errors was 77.2%. Prescription confirmation, physical injury, medication, and medical waste management errors were identified more frequently than surrounding environment and patient identification errors. Similar findings were reported by Kim (16), in which medication- and prescription-related errors were among the more frequently identified errors. This may be because medication administration, prescription checking, and visible physical safety hazards are repeatedly emphasized in nursing education. In contrast, students had greater difficulty identifying subtle environmental or patient condition-related errors. In particular, item 8, “patient was not wearing their hospital clothes properly,” and item 9, “bedsheets were soiled with patient’s urine,” had the lowest identification frequency. These findings suggest that nursing students without clinical practice experience may overlook environmental cues and patient-related details that can contribute to preventable safety incidents, including falls.

In our study, patient safety knowledge scores increased after the ROE simulation, and the adjusted posttest score was significantly higher in the experimental group than in the control group. These findings are consistent with a study by Kim et al. (8), who also reported increased patient safety knowledge scores after a simulation using patient safety case studies for nursing students. During the ROE simulation, students actively identified predefined patient safety errors and participated in an immediate debriefing session. The ROE simulation may have promoted active and experiential learning by allowing students to identify patient safety errors in a realistic clinical environment and immediately reflect on their observations during debriefing. Through direct observation, discussion, and reflection during the debriefing process, students may have developed greater awareness and confidence in applying patient safety principles in clinical practice. Debriefing is an important component of simulation-based learning because students analyze errors and develop understanding through discussion and reflection. While reflecting on their own practice during the debriefing process, students integrate their knowledge and experience (16, 27). The immediate debriefing may have helped students connect the identified errors with patient safety principles and reflect on their observations.

Patient safety attitude scores increased after the ROE simulation, and the adjusted posttest score was significantly higher in the experimental group than in the control group. In a study of medical college students, Madigosky et al. (28) also reported an overall increase in attitude scores after patient safety education. Previous studies have suggested that greater patient safety knowledge contributes to more positive attitudes toward patient safety (25, 29). However, other educational approaches, including flipped learning and case-based simulation, did not demonstrate significant improvements in patient safety attitudes (8, 10). These findings suggest that actively identifying patient safety errors in realistic clinical scenarios may be more effective in promoting positive patient safety attitudes than educational approaches relying primarily on theoretical instruction or case analysis. The ROE simulation encouraged students to actively identify patient safety hazards, discuss their observations with peers during debriefing, and reflect on appropriate patient safety practices. These learning activities may have increased students’ awareness of patient safety responsibilities, thereby contributing to more positive patient safety attitudes. Given that positive attitudes toward patient safety are associated with safe clinical behaviors, educational strategies that promote active engagement in patient safety learning may better prepare nursing students for clinical practice.

We observed a significant increase in patient safety performance confidence scores after the patient safety simulation. Stomski et al. (11) reported no change in patient safety performance confidence after situational awareness education, actually reporting a decrease in performance confidence after clinical practice. This indicates the importance of education methods for patient safety performance confidence in nursing students preparing for clinical practice. As situational awareness education by itself cannot sufficiently increase performance confidence, simulation education may be particularly valuable, as it allows students to learn through direct and indirect experiences. Students who begin clinical practice with low patient safety confidence may lose further confidence in front of patients, hindering their ability to receive effective practical education. These findings indicate that ROE simulation may be useful for strengthening performance confidence among nursing students preparing for clinical practice.

During the ROE simulation, students worked in teams to identify staged patient safety errors and participated in a debriefing session with experienced instructors. These team-based error identification and debriefing activities may have supported students’ reflection on patient safety risks and contributed to their improved performance confidence (30). Similarly, in the present study, actively identifying patient safety hazards, discussing them with peers, and reflecting on their performance during debriefing may have strengthened students’ confidence in applying patient safety principles in future clinical practice. Students with high performance confidence are more likely to solve patient safety-related problems rapidly and effectively (31). In a study by Gropelli and Shanty (1), students reported that they were afraid of making patient-related errors and that they would not report or ask about their own errors due to fear. These findings support the use of ROE simulations as an educational strategy to strengthen nursing students’ confidence in maintaining safe patient care environments before clinical practice.

The findings of this study suggest that the ROE simulation may serve as an effective educational strategy for improving nursing students’ patient safety knowledge, attitudes, and performance confidence before clinical practice. Nurses continually interact with patients; thus, they need to be able to recognize threats to patient safety faster than anyone else (32). Overall, the findings support the educational value of ROE simulation for nursing students preparing for clinical practice. ROE simulations may provide an effective experiential learning strategy to support the development of patient safety competencies among nursing students preparing for clinical practice.

### 4.1 Limitations

This study has several limitations. First, because a non-equivalent control group pretest-posttest design was used and participants were recruited from different institutions, caution is warranted when interpreting the findings, as unmeasured institutional differences may have influenced the results. Patient safety knowledge differed significantly between groups at baseline, possibly due to the non-equivalent design and unmeasured differences in prior patient safety-related learning experiences. Although this baseline difference was adjusted for in the ANCOVA, it should be considered when interpreting the intervention effect on patient safety knowledge. In addition, attrition in the control group resulted in unequal group sizes, which may have introduced attrition bias. Furthermore, because the control group did not receive an active comparison intervention, the specific effects of the ROE simulation should be interpreted with caution. Second, participants were recruited using convenience sampling, which may limit the generalizability of the findings. Third, because participants were not blinded to group allocation, the possibility of performance or response bias cannot be excluded. Fourth, the ROE simulation consisted of a single 45-min session and outcomes were assessed only immediately after the intervention; therefore, the retention of patient safety knowledge, attitudes, and performance confidence over time could not be determined. Finally, several outcome variables were measured using self-report questionnaires, which may have introduced response bias. Future studies should include follow-up assessments and use multicenter, randomized study designs with active comparison groups, where feasible, to evaluate the sustained and specific effects of ROE simulations.

## 5 Conclusion

This study evaluated the effects of a room of errors simulation on patient safety knowledge, attitudes, and performance confidence among nursing students preparing for clinical practice. Nursing students who participated in the ROE simulation showed greater improvements in patient safety knowledge, attitudes, and performance confidence than those in the control group. These findings suggest that ROE simulations may be a valuable educational strategy for strengthening patient safety education and preparing nursing students for safe clinical practice. Future room of errors interventions may be enhanced by including diverse environmental safety and patient identification scenarios and by using structured debriefing to reinforce less-recognized patient safety risks.

## Data Availability

The raw data supporting the conclusions of this article will be made available by the authors, without undue reservation.

## Footnotes

1
https://www.psychologie.hhu.de/en/arbeitsgruppen/allgemeine-psychologie-und-arbeitspsychologie/gpower
